# *In vitro* and *In Vivo* Drug Metabolism Analysis of BPI-460372 - A Covalent TEAD1/3/4 Inhibitor

**DOI:** 10.2174/0113892002351556250123105344

**Published:** 2025-02-11

**Authors:** Xiaoyun Liu, Dafang Zhong, Chongzhuang Tang, Xiaofeng Xu, Hong Lan, Xingxing Diao

**Affiliations:** 1 Betta Pharmaceuticals Co., Ltd, Hangzhou, 311100, China;; 2 Shanghai Institute of Materia Medica, Chinese Academy of Sciences, Shanghai, 201210, China;; 3 XenoFinder Co., Ltd, Suzhou 215123, China

**Keywords:** Hippo signaling pathway, TEAD inhibitor, covalent inhibitor, BPI-460372, UHPLC-Orbitrap-HRMS, cysteine S-conjugate β-lyase

## Abstract

**Background:**

BPI-460372 is an orally available, covalent, irreversible small molecule inhibitor of the transcriptional enhanced associate domain (TEAD) 1/3/4, which is currently in clinical development for the treatment of cancers with Hippo pathway alterations.

**Objectives:**

This study aimed to determine the cytochrome P450 (CYP) phenotyping, metabolic stability, and *in vitro* and *in vivo* metabolic profile of BPI-460372.

**Methods:**

The CYP phenotyping and metabolic stability were assessed by measuring the depletion of substrate. The metabolic profile in hepatocytes and rat and dog plasma was analyzed using ultra-high-performance liquid chromatography combined with Orbitrap tandem mass spectrometry (UHPLC-Orbitrap-HRMS).

**Results:**

BPI-460372 was mainly metabolized by CYP2D6, CYP3A4, and CYP1A2. BPI-460372 exhibited low clearance in human, monkey, and rat hepatocytes, while moderate clearance in dog and mouse hepatocytes. A total of 10 metabolites were identified in five species of hepatocytes, and no human-unique metabolite was detected. In rat plasma and dog plasma, the primary metabolites were M407 (BPI-460430) and M423 (BPI-460456), respectively. The two metabolites were quantitatively determined in rat and dog plasma in pharmacokinetic and toxicological studies. The major metabolic site was 2-fluoro-acrylamide, and major metabolic pathways in hepatocytes, and rat and dog plasma involved oxidative defluorination, hydration, glutathione (GSH) conjugation, hydrolysis, cysteine conjugation, and *N*-acetyl cysteine conjugation. *β*-lyase pathway contributed to the metabolism of BPI-460372 in rats to a certain degree.

**Conclusion:**

This study elucidated the metabolism of BPI-460372 and provided a basis for pharmacokinetic and toxicological species selection, human pharmacokinetics prediction, and assessment of clinical co-administration limitations and possible metabolic pathways in humans.

## 1 INTRODUCTION

The Hippo signaling pathway consists of a series of conserved kinases found in drosophila and is involved in some biological functions, including tissue development, maintenance of tissue homeostasis, and regenerative repair [1]. Yes-associated protein (YAP) and transcriptional coactivator with PDZ-binding motif (TAZ) are two important transcriptional coactivators in the Hippo pathway, which regulate the transcription of target genes by binding to the TEAD1-4 to form the YAP/TAZ-TEAD complex [2]. Dysregulation of the Hippo pathway leads to tumor cell development, migration, and drug resistance, and YAP and TAZ are hyperactivated in a variety of solid tumors, including lung, colorectal, breast, pancreatic, hepatocellular, melanoma, and glioma [3]. Given the critical role of the YAP/TAZ-TEAD complex in tumorigenesis, the YAP/TAZ-TEAD complex has become an attractive target for the development of anticancer drugs. Some molecules target the YAP/TAZ-TEAD complex and some molecules directly target the palmitoylation pocket of TEAD [4]. For TEAD small molecule inhibitors, no drug has been approved and only four molecules have entered clinical trials, namely VT3989 (NCT04665206), IAG933 (NCT04857372), BPI-460372 (NCT05789602), and ODM-212[5]. BPI-460372 is a covalent and irreversible TEAD inhibitor, unlike VT3989 and IAG933, which are non-covalent and reversible TEAD inhibitors [6, 7]. Fig. (**1**) shows the chemical structure of BPI-460372. A phase I clinical trial (NCT05789602) has been conducted in China to assess the safety, tolerability, pharmacokinetics, and preliminary efficacy of BPI-460372 in solid tumor patients. BPI-460372 exhibited a good safety profile, and preliminary efficacy was observed in patients with malignant mesothelioma, lung adenocarcinoma, and lung squamous cell carcinoma [8]. The metabolism profile of BPI-460372 was unknown previously and has been characterized in a series of preclinical and *in vitro* and *in vivo* studies.

This study aimed to (i) identify the major metabolic enzymes involved in the metabolism of BPI-460372, (ii) evaluate the metabolism stability and profile of BPI-460372 in hepatocytes of different species, and (iii) elucidate the metabolic profile and pathway of BPI-460372 in rat and dog plasma. The findings could generate more reliable biotransformation information on BPI-460372 and provide evidence to elucidate potential drug-drug interaction (DDI) mechanisms.

## 2 MATERIALS AND METHODS

### 2.1 Materials

BPI-460372, BPI-460430, BPI-460456, and BPI-460608 were provided by Betta Pharmaceuticals Co., Ltd. (Hangzhou, China). The following chemicals were purchased from Sigma-Aldrich (Poole, Dorset, UK): phenacetin, diclofenac, terfenadine, tolbutamide, α-naphthoflavone, ticlopidine, quercetin, sulfaphenazole, ketoconazole, high-performance liquid chromatography (HPLC)-grade acetonitrile and methanol, and nicotinamide adenine dinucleotide phosphate (NADPH). Bupropion and midazolam were supplied by the National Institutes for Food and Drug Control (Beijing, China). Paclitaxel and *S*-mephenytoin were obtained from J&K Scientific (Beijing, China). Quinidine and formic acid were purchased from Adamas (Basel, Switzerland). Dextromethorphan, 7-ethoxycoumarin (7-EC), and testosterone were purchased from Toronto Research Chemicals Inc. (North York, Canada), Dalian Meilun Biotechnology Co., Ltd. (Dalian, China), and Aladdin (Shanghai, China), respectively. Aminooxyacetic acid (AOAA) was purchased from Shanghai Macklin Biochemical Technology Co., Ltd (Shanghai, China). Ultrapure water was prepared by the Milli-Q system (Molsheim, France). Recombinant human CYP isozymes *(e.g.*, 1A2, 2A6, 2B6, 2C8, 2C9, 2C19, 2D6, 2E1, 3A4, and 3A5) were purchased from Cypex Ltd. (Dundee, UK). Pooled human liver microsomes (HLMs) were obtained by BioIVT (Baltimore, MD, USA). Primary hepatocytes of human, cynomolgus monkey, beagle dog, Sprague-Dawley (SD) rat, and Institute of Cancer Research (ICR) mouse were provided by XenoTech (Lenexa, KS, USA).

### 2.2 Animals

SD rats and beagle dogs were supplied by Beijing Vital River Laboratory Animal Technology Co., Ltd. (Beijing, China) and Changzhou Beile Experimental Animal Breeding Co., Ltd. (Changzhou, China), respectively.

### 2.3 Metabolic Stability of BPI-460372 in HLMs with or without Specific CYP Inhibitors

Before starting the experiments, the HLMs were thawed gently on ice. Metabolic stability incubations were performed at 37 °C. The incubations contained 100 mM phosphate buffer saline (PBS, pH 7.4), HLMs (1 mg protein·mL^-1^), dimethyl sulfoxide (DMSO, blank control) or a selective CYP inhibitor, and BPI-460372 or positive control substrates (final incubation concentration, 1 μM). The incubation process started with the addition of NADPH (1 mM final concentration) after a 5-minute preincubation period. The chemical inhibitors were as follows: a-naphthoflavone (2 µM) for CYP1A2, ticlopidine (20 µM) for 2B6, quercetin (20 µM) for CYP2C8, sulfaphenazole (20 µM) for CYP2C9, ticlopidine (200 µM) for CYP2C19, quinidine (20 µM) for CYP2D6, and ketoconazole (20 µM) for CYP3A [9]. Aliquots were maintained in acetonitrile containing internal standard (IS) at various time points, such as 0 min, 5 min, 10 min, 20 min, 30 min, and 60 min. All incubations were performed in triplicate, and the concentration in the supernatant was determined by liquid chromatography-tandem mass spectrometry (LC-MS/MS).

The elimination rate constant k (min^-1^) was obtained by linear regression from the natural logarithm of the elimination percentage of the parent drug and the incubation time. The inhibition rate was calculated using equation 1 [10]:


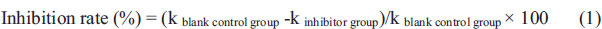


### 2.4 Metabolic Stability of BPI-460372 in Recombinant Human CYP Isoenzymes

To identify the specific isoform that participates in the metabolism of BPI-460372, 1 µM BPI-460372 or positive control substrates were mixed with recombinant human CYP1A2, 2B6, 2C8, 2C9, 2C19, 2D6 or 3A4 (100 pmol P450·mL^-1^), and the reaction was started by the addition of NADPH (1 mM final concentration) after 5-minute preincubation at 37°C. Aliquots were maintained in acetonitrile containing IS at different time points at 0 min, 5 min, 10 min, 20 min, 30 min, and 60 min. All incubations were performed in triplicate, and the concentration of the supernatant was determined by LC-MS/MS.

A substrate depletion approach was employed to determine the intrinsic clearance (CL_int (rCYPj)_) for each isoform. The contribution of each isoform was estimated using equations 2 and 3. CYP_j_ abundance was evaluated as the abundance (CYP_j_) of each isoform in HLMs (pmol/mg protein) [11].


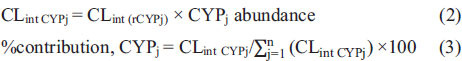


### 2.5 Metabolic Stability of BPI-460372 in Human, Monkey, Dog, Rat, and Mouse Hepatocytes

1 µM BPI-460372 or positive control (testosterone and 7-EC) was incubated in triplicate with cryopreserved hepatocytes (1 × 10^6^ cells·mL^-1^) at 37 °C in 5% CO_2_ atmosphere. After mixing, 20 μL of cell suspension was serially transferred into 200 μL of cold acetonitrile containing IS at the time points of 0, 5, 15, 30, 60, and 120 min, respectively. For the negative control samples from inactive hepatocytes (boiled in the water bath at 100°C for 10 min to eliminate enzymatic activity), 20 μL of cell suspension was serially transferred into 200 μL of chilled acetonitrile containing IS at the time points of 0 and 120 min. The samples were analyzed by LC-MS/MS and then semi-quantified using the peak area ratio of analyte versus IS.

The metabolic stability was assessed by measuring the depletion of substrate. The half-life (*t*_1/2_), intrinsic clearance (CL_int_, *_in virto_*, µL·min^-1^·million cells^-1^), whole liver intrinsic clearance (CL_int_, *in vivo*, mL·min-1·kg^-1^), and *in vivo* hepatic clearance (CL_H_, mL·min-1·kg^-1^) were calculated following a reported method [12]. In the calculation of CL_H_, the fraction drug unbound (F_ub_) was set as 1. The hepatic blood flow, liver weight, and hepatocellularity of five species were used for the calculation of CL_int_, *_in vivo_* [13, 14]. The hepatic extraction ratio (E_h_) means the ratio of CL_H_ to hepatic blood flow.

### 2.6 Metabolism and Metabolite Formation in Human, Monkey, Dog, Rat, and Mouse Hepatocytes

10 µM BPI-460372 was incubated in five species (human, cynomolgus monkey, beagle dog, SD rat, and ICR mouse) hepatocytes (1 × 10^6^ cells·min^-1^) at 37 °C for 4 h. 7-EC, as positive control, was incubated for 0 h and 4 h. For blank control, hepatocytes were inactivated by adding ice-cold acetonitrile, and then the BPI-460372 solution was added. All the samples were duplicates. Incubations were stopped using the same volume of cold acetonitrile. The tube was vortexed, and the duplicate samples were combined. Metabolite profiling and identification of BPI-460372 were conducted by using UHPLC-Orbitrap-HRMS. The samples of 7-EC were analyzed by LC-MS/MS and then semi-quantified using the peak area ratio of analyte versus IS.

### 2.7 Metabolite Identification in SD Rats and Dogs Plasma

The plasma samples were acquired from SD rats (*n*=6, 3 female and 3 male) and beagle dogs (*n*=6, 3 female and 3 male) after administration of 12 mg/kg and 8 mg/kg of BPI-460372, respectively. The rat urine, feces, and bile samples were acquired in SD rats after administration of 6 mg/kg of BPI-460372.

The plasma samples of 3 female and 3 male rats at pre-dose, 0.25, 0.5, 1, 2, 4, 6, 8, 10, and 24 h post-dose were selected and pooled according to the area under the curve (AUC)-pooling principle for individuals [15]. Equal volume from each rat was pooled together to get 1 male and 1 female 0-24 h plasma samples. An equal percentage by volume of individual urine sample (0-8, 8-24, and 24-48 h) from 3 female and 3 male rats was pooled by time interval across rats, respectively, resulting in two pooled urine samples (1 male and 1 female). An equal percentage by weight of individual feces samples (0-8, 8-24, and 24-48 h) was pooled by time interval across female and male rats, resulting in two pooled fecal samples. An equal percentage by volume of individual bile samples (0-4, 4-8, 8-24, and 24-48 h) was pooled by time interval across female and male rats, resulting in two pooled bile samples. The plasma samples of 3 female and 3 male dogs at pre-dose, 0.25, 0.5, 1, 2, 4, 6, 8, 10, 24, and 48 h post-dose were selected and pooled according to the AUC-pooling principle for individuals [15]. Equal volumes from 3 female and 3 male rats were pooled together to get two 0-48 h plasma samples. For blank samples of plasma, urine, and bile, equal volume was pooled across subjects. For a blank sample of feces, equal weight was pooled across rats.

The pooled plasma, feces, and bile samples were extracted by adding 3-fold volume of acetonitrile, and the mixture was vortexed (1 min) and sonicated (1 min) before centrifugation (7220 g, 5 min). The pooled urine samples were centrifuged (7220 g, 5 min). Then the supernatant was transferred into a clean tube and evaporated to dryness under the nitrogen stream. The residues were redissolved with an appropriate volume of acetonitrile/water (20: 80, *v: v*). After centrifugation, the redissolved solution was transferred into an HPLC vial for UHPLC-Orbitrap-HRMS (15000 g, 10 min, 4°C).

### 2.8 The Effect of Cysteine S-conjugate β-lyase Inhibitor on the Metabolism of BPI-460372

To investigate the effect of cysteine *S*-conjugate *β*-lyase on the metabolism of BPI-460372, SD rats were treated with 20 mg/kg BPI-460372 alone (n=3, male) or 20 mg/kg BPI-460372 coadministration with 100 mg/kg cysteine *S*-conjugate *β*-lyase inhibitor AOAA (n=3, male), once daily for 21 consecutive days. On day 21, urine samples were collected at 0-8, 8-24 and 24-48 h. The same percentage of urine samples from each rat was taken at these three time intervals and mixed. The samples were analyzed by LC-MS/MS and then semi-quantified using the peak area ratio of analyte versus IS.

### 2.9 LC-MS/MS Detection

For the CYP phenotyping study, BPI-460372 was quantitated by a Sciex ExionLC AD liquid chromatograph coupled with a Triple Quad 6500+ MS (AB Sciex, Singapore). For hepatocyte metabolic stability, BPI-460372 was detected using an ACQUITY UPLC I-Class Plus system (Waters, Milford, MA, USA) coupled with a Triple Quad 6500+ MS (AB Sciex, Framingham, MA, USA). For the effect of cysteine *S*-conjugate *β*-lyase inhibitor on the metabolism of BPI-460372 study, the metabolites of BPI-460372 were analyzed using a Sciex ExionLC AD liquid chromatograph coupled with a Triple Quad 4500 MS (AB Sciex, Singapore). Data acquisition and processing were carried out using Analyst software version 1.7.1 (AB Sciex). The detailed LC-MS/MS and LC/MS methods are displayed in the **supplementary material** section.

### 2.10 UHPLC-Orbitrap-HRMS Detection

The UHPLC-Orbitrap-HRMS analysis was carried out using a Vanquish UHPLC system (Thermo, Waltham, MA, USA), Thermo Q-Exactive Plus Hybrid Quadrupole Orbitrap Mass spectrometer equipped with an ESI interface operating in positive ion mode.

For hepatocyte metabolite identification, the different metabolites of BPI-460372 were detected using an ACQUITY UPLC HSS T3 column (1.8 µm, 100 mm × 2.1 mm) and ACQUITY UPLC BEH C18 column (1.7 µm, 100 mm × 2.1 mm) for rat and dog metabolite identification. The mobile phase contained a mixture of 0.1% formic acid in water (A) and 0.1% formic acid in acetonitrile (B). The gradient elution program was as follows: 0.0-1.0 min, 5% B; 1.0-7.0 min, 5%-30% B; 7.0-12.0 min, 30%-80% B; 12.0-16.0 min, 80%-95% B; 16.0-18.0 min, 95% B; 18.0-18.1 min, 95%-5% B. Then, 5% B from 18.1 to 20.0 min was maintained for equilibration. The flow rate was set to 0.5 mL/min, and the column temperature was kept at 40°C. Eluted fractions were monitored by a UV detector at 314 nm.

MS parameters were optimized as follows: spray voltage 3.5 kV; capillary temperature 320°C; sheath gas flow rate 40 L/h; and auxiliary gas flow rate 10L/h. MS data were acquired under the centroid mode within an *m/z* range of 100-1500 Da (full mass - MS/MS scan). The collision energy was 35, 45, and 60 V. The mass resolutions for full mass scan and MS/MS scan were 35000 and 17500, respectively.

Data acquisition was performed using Xcalibur software (Thermo). Data were analyzed using Compound Discoverer software (Thermo). The molecular formula of the metabolite was determined based on the accurate molecular mass of the metabolite. Based on the BPI-460372 mass spectral fragmentation pattern, we compared the metabolites' fragmentation information with the parent and speculated the possible metabolic sites or ranges of metabolites, and each metabolite was named using the letter ‘M’ followed by its molecular weight [16].

## 3 RESULTS

### 3.1 CYP Reaction Phenotyping of BPI-460372

Using HLMs and recombinant CYP enzyme to determine the CYP reaction phenotyping of BPI-460372, the results showed that BPI-460372 was mainly metabolized by CYP2D6, CYP3A4 and CYP1A2 (Table **1**). Quercetin was not the specific inhibitor of CYP2C8 and could also inhibit CYP2C19, CYP3A4, CYP2D6, and carboxylesterase to a certain extent [17, 18]. Therefore, the results of CYP2C8 reaction phenotyping of BPI-460372 only referred to the data of recombinant CYP enzyme. The data of positive control substrates indicated the experimental condition as reliable (Table **S1**).

### 3.2 Metabolic Stability of BPI-460372 in Human, Monkey, Dog, Rat, and Mouse Hepatocytes

The E_h_ of BPI-460372 in human, monkey, dog, rat, and mouse hepatocytes was 0.115, 0.253, 0.446, 0.200, and 0.406, respectively (Table **2**). In human, monkey, and rat hepatocytes, the E_h_ of BPI-460372 was less than 0.3, indicating low clearance. In dog and mouse hepatocytes, the E_h_ of BPI-460372 was between 0.3 and 0.7, which indicated moderate clearance. The remaining percentage of BPI-460372 in human, monkey, dog, rat, and mouse hepatocytes after 120 min incubation was 97.3%, 61.7%, 66.6%, 72.4%, and 54.7%, respectively. The t*_1/2_* values of testosterone and 7-hydroxycoumarin were in a range of 5-30 min and 10-50 min, respectively, meeting the acceptance criteria (Table **S2**).

### 3.3 Metabolism and Metabolite Formation in Human, Monkey, Dog, Rat, and Mouse Hepatocytes

**Fig. (2):**
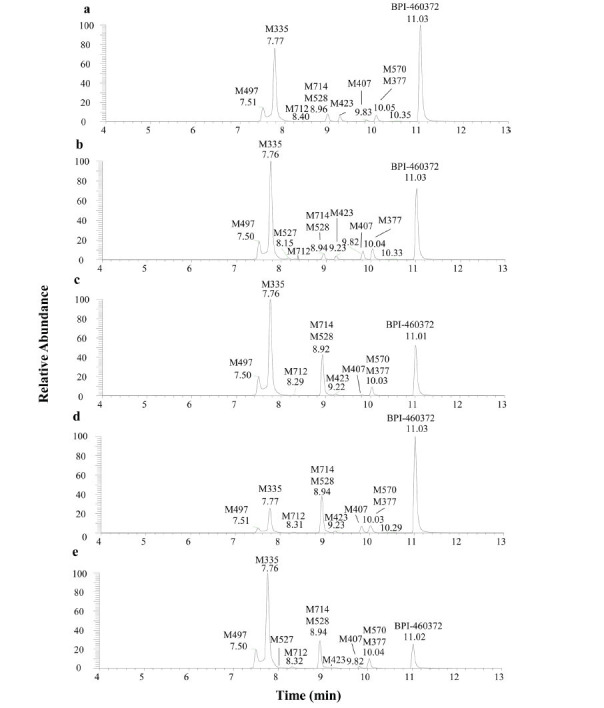
The extracted ion chromatograms of BPI-460372 and its metabolites from human (**a**), monkey (**b**), dog (**c**), rat (**d**), and mouse (**e**) hepatocytes.

**Fig. (3):**
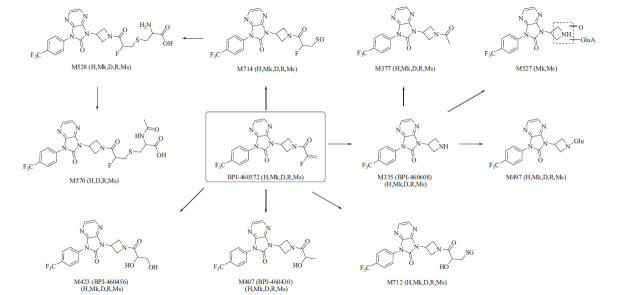
Proposed metabolic pathways of BPI-460372 in human (H), monkey (Mk), dog (D), rat (R), and mouse (Ms) hepatocytes.

A total of 10 metabolites of BPI-460372 were identified in five species of hepatocytes, and the extracted ion chromatograms (XICs) of metabolites provided an overall metabolite profile of BPI-460372 from five species (Fig. **2**). BPI-460372 and metabolites were detected by UV (λ = 314 nm), and BPI-460372 was the main drug-related component, with the relative abundance accounting for 76.96%, 63.98%, 48.28%, 70.87%, and 32.74% of total drug-related components in human, monkey, dog, rat, and mouse hepatocytes, respectively (Table **3**). The data of 10 detected metabolites, including metabolic pathway, retention time, formula, observed and theoretical *m/z*, mass error, and fragment ions are summarized (Table **4**), and the proposed structures of the 10 metabolites are illustrated (Fig. **3**). The major metabolic pathways of BPI-460372 in five species hepatocytes involved hydrolysis, hydration, glucose conjugation, oxidation, glucuronidation, GSH conjugation, oxidative defluorination, cysteine conjugation, *N*-acetylcysteine conjugation, and acetylation. The metabolites identified in human hepatocytes were also detected in animal hepatocytes, and no human-unique metabolite was detected.

The metabolite 7-EC-GluA, which was the de-ethylation and glucuronidation metabolite of 7-EC, was detected in hepatocytes of five species (Table **S3**). Thus, the enzymatic activity in phase I and phase II of all hepatocytes used was qualified.

The chromatographic behaviors and MS fragmentation patterns of BPI-460372 and its metabolites PI-460430, BPI-460456, and BPI-460608 were first assessed to identify other potential metabolites. The fragment ions and tentative fragmentation patterns of BPI-460372, BPI-460430, BPI-460456, and BPI-460608 are illustrated (Figs. **S1**-**4**).

### 3.4 Metabolite Identification in Rat Plasma, Urine, Feces, and Bile

A total of 17 metabolites were identified in rat plasma, urine, feces, and bile (Table **5**). In rat plasma, unchanged BPI-460372 was the most abundant component, accounting for 75.36% of total drug-related components (Table **6**). M335 (BPI-460608), M423 (BPI-460456), and M407 (BPI-460430) were the most abundant (MS peak area) metabolites, accounting for 2.48%, 2.71%, and 16.39% of total drug-related components, respectively (Table **6**). In rat urine, M570, M423 (BPI-460456), and M453 were the most abundant metabolites, accounting for 34.25%, 17.63%, and 17.25% of total drug-related components, respectively (Table **6**). In rat feces, M389, M391, and unchanged BPI-460372 were the most abundant components, accounting for 16.59%, 21.50%, and 16.95% of total drug-related components, respectively (Table 6). In rat bile, downstream products of GSH-conjugated metabolites M528 and M570 were the most abundant components, accounting for 43.88% and 45.40% of total drug-related components, respectively (Table **6**). The major metabolic pathways in rats involved oxidative defluorination, hydration, GSH conjugation, hydrolysis, cysteine conjugation, and *N*-acetyl cysteine conjugation, and the proposed metabolic pathways of BPI-460372 in rats are presented in Fig. (**4**).

### 3.5 Metabolite Identification in Dog Plasma

A total of 9 metabolites were identified in dog plasma (Table **6**). Unchanged BPI-460372, M423 (BPI-460456), and M335 (BPI-460608) were the most abundant components, accounting for 30.93%, 40.81%, and 8.45% of total drug-related components, respectively (Table **6**). The major metabolic pathways in dog plasma involved oxidative defluorination, hydration, hydrolysis, cysteine conjugation, and reductive defluorination. The proposed metabolic pathways of BPI-460372 in dog plasma are presented in Fig. (**5**).

### 3.6 The Effect of Cysteine *S*-conjugate *β*-lyase Inhibitor on the Metabolism of BPI-460372

In this study, upon administration of 20 mg/kg BPI-460372 alone or coadministration with 100 mg/kg AOAA once daily for 21 consecutive days in SD rats, BPI-460372 and metabolites M453, M528, M568, M570, M335, and M423 were detected in rat urine. The area ratio of mercapturate and *β*-lyase pathway metabolites (M453, M528, M568, and M570) was reduced when BPI-460372 was coadministrated with AOAA (Table **7**), which indicated the *S*-conjugate *β*-lyase to participate in the metabolism of BPI-460372.

## 4 DISCUSSION

The CYP enzyme phenotyping study revealed BPI-460372 to be predominantly metabolized by CYP2D6 (43.6%), followed by CYP3A4 (28.3%), CYP1A2 (13.1%), CYP2C8 (8.8%), and CYP2C9 (6.3%). In a phase I clinical study, to avoid the potential DDI, trial participants were not allowed to use inhibitors of CYP1A2, CYP3A4, and CYP2D6 or inducers of CYP1A2 and CYP3A4 for seven days before and during the clinical study.

The metabolic stability study indicated BPI-460372 to have low clearance in human, monkey, and rat hepatocytes, and median clearance in mouse and dog. BPI-460372 exhibited low clearance in rats and monkeys, and median clearance in dogs *in vivo*, showing good *in vitro*-*in vivo* correlation. According to the metabolic stability of BPI-460372 in human hepatocytes, the clearance in humans was predicted to below.

Our study showed that the metabolites identified in human hepatocytes were also detected in animal hepatocytes, and no human unique metabolite was detected. Based on the result, rats and dogs were chosen as the species for the pharmacokinetic and toxicological study. For BPI-460372, the main metabolic pathway included (1) hydrolysis (M335), followed by glucose conjugation (M497), mono-oxidation and glucuronidation (M527), and acetylation (M377); (2) GSH conjugation (M714), followed by metabolism to form *N*-acetylcysteine conjugation (M570) and cysteine conjugation (M528). Oxidative defluorination and hydration also participated in the metabolism of BPI-460372. Without the addition of NADPH, the formation of M335 after 45 minutes of incubation of BPI-460372 with HLMs was comparable to that of the HLMs+NADPH group, suggesting the other non-CYP enzymes, such as esterases, to be involved in the production of M335.

In rat and dog plasma, the primary metabolites were M407 (BPI-460430) and M423 (BPI-460456), respectively. The two metabolites were quantitatively determined in rat and dog plasma in pharmacokinetic and toxicological studies.

BPI-460372 contained a modified electrophilic functionality (“warhead”) 2-fluoro acrylamide, which could covalently bind to the Cys-368 residue of TEAD1/3/4 without significant off-target covalent binding affinity and inhibit the palmitoylation of TEADs [19]. All metabolites detected in hepatocytes and rat and dog plasma were more polar than the parent drug. The 2-fluoro acrylamide was the main metabolism site that inactivated during metabolism, except for M389, which contained acrylamide. *In vitro* studies have shown metabolites BPI-460608, BPI-460456, and BPI-460430 to be inactive metabolites that do not covalently bind to TEAD proteins or inhibit TEAD palmitoylation. Adagrasib is a small molecule inhibitor of KRAS G12C mutant isoform indicated for the treatment of adult patients with KRAS G12C-mutated locally advanced or metastatic non-small cell lung cancer (NSCLC). Adagrasib contained 2-fluoro-acrylamide and was subjected to extensive metabolism in animals and humans [20]. Compared to the acrylamide counterpart, 2-fluoro-acrylamide electrophile showed less reactivity toward GSH [21]. Adagrasib (2-fluoro-acrylamide warhead) showed higher stability in mouse, dog, and human whole blood, and a much reduced glutathione-*S*-transferase (GST)-mediated GSH conjugation than compound 18 (acrylamide warhead) [22].

GSH is a nucleophile, which could form GSH conjugate with target covalent inhibitors (TCI), and serve as a route of metabolism *in vivo* of TCI. GST enzymes can accelerate the addition reaction by catalysis, and GST has been found to be expressed in the liver, spleen, and extrahepatic tissues [23]. The metabolism of GSH conjugate is called the mercapturic acid pathway [24]. GSH conjugates could be further metabolized by γ-glutamyl transpeptidase (GGT) and cysteinylglycine dipeptidase to form cysteine-glycine conjugate and cysteine conjugate, respectively. Cysteine conjugate can be further acetylated by *N*-acetyltransferase to form the mercapturic acid conjugate (*N*-acetylcysteine conjugate), and the reaction occurs primarily in the kidney. Cysteine conjugate could also be cleaved by cysteine *S*-conjugate *β*-lyase to release a free thiol. Cysteine *S*-conjugate *β*-lyase is present in high concentrations in the kidney [24]. The free thiol can be catalysed by thiol methyltransferase and UDP-glucuronosyltransferases to form methylthio derivative and glucuronyl *S*-conjugate, respectively [25].

The high reactivity of free thiol can lead to covalent modification of macromolecules, depletion of nonprotein thiols, lipid peroxidation, and carbonylation of susceptible proteins, leading to toxicity. The renal proximal tubule has a high metabolic rate, and free thiol inactivates key enzymes in the tricarboxylic acid (TCA) cycle, resulting in mitochondrial toxicity and thus renal proximal tubule injury [25]. Because of greater specific activities of cysteine *S*-conjugate *β*-lyase in rats than in humans, the rat renal tissue is more susceptible than that of humans to renal toxicity caused by cysteine *S*-conjugate *β*-lyase-mediated bioactivation. Some relevant examples of species-dependent metabolism leading to nephrotoxicity include efavirenz, hexachlorobutadiene, and sotorasib [26].

In rats, the mercapturic acid and *β*-lyase pathway metabolites accounted for 60.72% and 97.95% of the total drug-related material in urine and bile, respectively. The result indicated that the mercapturic acid and *β*-lyase pathway contributed to the metabolism of BPI-460372 in rats to a certain degree. In the 4-week toxicity study of BPI-460372, the main target organs of rats included the kidney. Our study indicated that the cysteine *S*-conjugate *β*-lyase participated in the metabolism of BPI-460372, and the relationship between the nephrotoxicity observed in rats and cysteine *S*-conjugate *β*-lyase-mediated bioactivation needs further study.

Acrylamide warheads are widely used in covalent tyrosine kinase inhibitors (TKIs), and 8 covalent TKIs currently approved by the FDA include acalabrutinib, afatinib, dacomitinib, ibrutinib, neratinib, osimertinib, ritlecitinib, and zanubrutinib [27]. For acalabrutinib, dacomitinib, ibrutinib, neratinib, and zanubrutinib, hepatic metabolism *via* CYPs is the major route of elimination [28-32]. Ritlecitinib is metabolized through multiple CYPs and GSTs pathways with no single route contributing more than 25% of the total metabolism [33]. For afatinib and osimertinib, the main pathway of clearance is covalently binding to plasma proteins [34, 35]. It has been reported that a variety of covalent TKIs can covalently bind to human serum albumin, which leads to its instability in human plasma [36]. BPI-460372 was stable when incubated with mouse, rat, dog, monkey, and human plasma at 37°C for 4 h, indicating that it did not covalently bind to plasma proteins.

## CONCLUSION

BPI-460372 was mainly metabolized by CYP2D6, CYP3A4, and CYP1A2, and to avoid potential clinically pharmacokinetic DDI, inducers or inhibitors of related enzymes were limited in phase I clinical study. The good *in vitro*-*in vivo* correlation of clearance is favorable for predicting the pharmacokinetics of BPI-460372 in humans. The *in vitro* and *in vivo* studies here have provided important information on the metabolism of BPI-460372. Further clinical development of BPI-460372 is ongoing.

## Figures and Tables

**Fig. (1):**
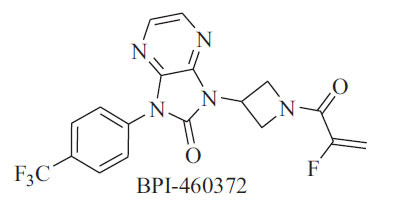
The chemical structure of BPI-460372.

**Fig. (4):**
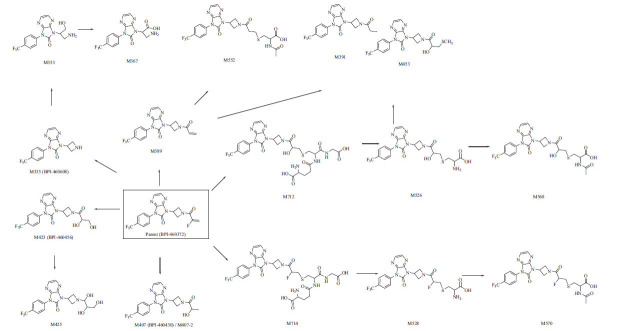
Proposed metabolic pathways of BPI-460372 in rats.

**Fig. (5):**
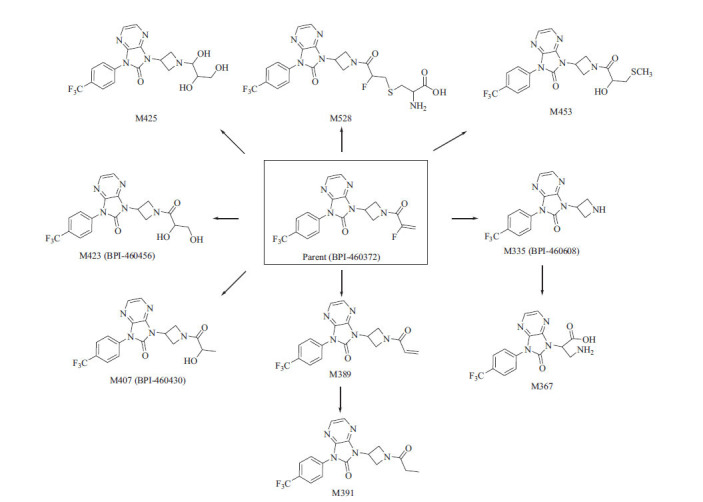
Proposed metabolic pathways of BPI-460372 in dog plasma.

**Table 1:** The inhibition rate of metabolism of BPI-460372 in HLM by specific CYP inhibitors and percentage of metabolism of BPI-460372 through recombinant expressed cytochrome P450 isoforms.

**CYPs**	**Inhibition Rate (%)**	**Contribution (%)**
1A2	32.7	13.1
2B6	-11.5	NC^a^
2C8	53.9	8.8
2C9	NC^b^	6.3
2C19	-4.2	NC^a^
2D6	17.0	43.6
3A4	9.1	28.3

**Note:** ^a^BPI-460372 showed no metabolic trend upon incubation with rCYP2B6 and 2C19, thus the k and contribution were not calculated. ^b^BPI-460372 had no metabolic trend in liver microsomes with CYP2C9 inhibitor, thus the k and inhibition rate were not calculated. **Abbreviation:** NC, not calculated.

**Table 2:** Metabolic stability results of BPI-460372 in human, monkey, dog, rat, and mouse hepatocytes.

**Species**	**t*_1/2_* (min)**	**CL_int_, *_ in virto_* (µL·min^-1^·Million cells^-1^)**	**CL_int_, *_ in vivo_* (mL·min^-1^·kg^-1^)**	**CL_H_ (mL·min^-1^·kg^-1^)**	**E_h_**
Human	928.6	0.746	2.7	2.4	0.115
Monkey	167.2	4.145	14.9	11.1	0.253
Dog	191.5	3.619	24.9	13.8	0.446
Rat	235.7	2.941	13.8	11.0	0.200
Mouse	133.6	5.187	61.6	36.6	0.406

**Table 3:** UV (λ=314 nm) relative abundance of BPI-460372 and its metabolites in human, cynomolgus monkey, beagle dog, SD rat, and ICR mouse hepatocytes after 4 h incubation (%).

**ID**	**Retention Time**	**UV (314 nm) Peak Areas Relative Abundance (%)**				
	**(min)**	**Human**	**Monkey**	**Dog**	**Rat**	**Mouse**
BPI-460372	11.02	76.96	63.98	48.28	70.87	32.74
M497	7.5	4.71	6.54	7.81	1.48	9.7
M335 (BPI-460608)	7.76	11.47	16.99	18.82	3.9	26.4
M527	8.16	ND	1.35	ND	ND	+
M712	8.32	+	+	+	+	1.43
M714	8.94	3.01	3.01	22.65	18.4	24.76
M528						
M423 (BPI-460456)	9.23	2.36	2.01	+	+	+
M407 (BPI-460430)	9.82	+	3.61	+	2.75	1.39
M570	10.01	1.48	ND	2.44	2.6	3.57
M377			2.50			

**Note:** ND, not detected by UV and MS; +, The UV signal was too weak to be quantified, but could be detected in MS.

**Table 4:** Characterization of BPI-460372 metabolites in human, cynomolgus monkey, beagle dog, SD rat and ICR mouse hepatocytes through UHPLC-Orbitrap-HRMS.

**Name**	**Metabolic Pathway**	**Retention Time (min)**	**Formula**	**Observed (*m/z*)**	**Theoretical (*m/z*)**	**Mass Error (ppm)^a^**	**Fragment Ions**
M497	Hydrolysis and glucose conjugation	7.50	C_21_H_22_F_3_N_5_O_6_	498.1591	498.1595	-0.8	480.1489, 462.1284, 378.1172, 336.1067, 307.0801, 281.0645, 174.0774, 98.0600
M335 (BPI-460608)	Hydrolysis	7.76	C_15_H_12_F_3_N_5_O	336.1060	336.1067	-2.1	307.0801, 281.0645, 238.0587, 97.0396, 56.0495
M527	Hydrolysis, mono-oxidation and glucuronidation	8.16	C_21_H_20_F_3_N_5_O_8_	528.1329	528.1337	-1.5	352.1016, 323.0750, 322.0910, 281.0645, 238.0587
M712	Oxidative defluorination (-F+OH) and glutathione conjugation	8.32	C_28_H_31_F_3_N_8_O_9_S	713.1951	713.1960	-1.3	638.1639, 464.0999, 438.0842, 390.1172, 319.0801, 307.0801, 281.0645
M714	Glutathione conjugation	8.94	C_28_H_30_F_4_N_8_O_8_S	715.1908	715.1916	-1.1	640.1601, 440.0799, 408.1078, 390.1172, 336.1067, 319.0801, 281.0645
M528	Cysteine conjugation	8.94	C_21_H_20_F_4_N_6_O_4_S	529.1269	529.1276	-1.3	440.0799, 408.1078, 390.1172, 336.1067, 319.0801, 307.0801, 281.0645, 56.0495
M423 (BPI-460456)	Oxidative defluorination (-F+OH) and hydration	9.23	C_18_H_16_F_3_N_5_O_4_	424.1219	424.1227	-1.9	406.1122, 336.1067, 319.0801, 307.0801, 281.0645, 56.0495
M407 (BPI-460430)	Defluorination (-F+H) and hydration	9.82	C_18_H_16_F_3_N_5_O_3_	408.1269	408.1278	-2.2	364.1016, 336.1067, 319.0801, 307.0801, 281.0645, 56.0495
M570	*N*-acetylcysteine conjugation	10.01	C_23_H_22_F_4_N_6_O_5_S	571.1371	571.1381	-1.8	529.1276, 440.0799, 408.1078, 336.1067, 319.0801, 307.0801, 281.0645, 162.0219
M377	Hydrolysis and acetylation	10.01	C_17_H_14_F_3_N_5_O_2_	378.1165	378.1172	-1.9	337.0907, 336.1067, 319.0801, 307.0801, 281.0645, 238.0587, 97.0396, 56.0495
BPI-460372	Parent drug	11.02	C_18_H_13_F_4_N_5_O_2_	408.1071	408.1078	-1.7	319.0801, 307.0801, 281.0645, 238.0587, 128.0506, 102.0350, 97.0396, 73.0084

**Note:** ^a^Mass error = (Observed *m/z* – Theoretical *m/z*) / Theoretical *m/z*; 1 ppm = 1 × 10^-6^.

**Table 5:** Characterization of BPI-460372 metabolites in rat plasma, urine, feces, bile, and dog plasma through UHPLC-Orbitrap-HRMS.

**Name**	**Metabolic Pathway**	**Retention Time (min)**	**Formula**	**Observed (*m/z*)**	**Theoretical (*m/z*)**	**Mass Error (ppm)^a^**	**Fragment Ions**
BPI-460372	Parent drug	10.65	C_18_H_13_F_4_N_5_O_2_	408.1076	408.1078	-0.6	319.0801, 307.0801, 281.0645, 238.0587, 128.0506, 102.0350, 97.0396, 73.0084
M367	Oxidation (+2O) of BPI-460608	6.53	C_15_H_12_F_3_N_5_O_3_	368.0959	368.0965	-1.7	351.0700, 339.0700, 307.0801, 293.0645, 281.0645, 97.0396
M353	Oxidation (+O) and hydrogenation of BPI-460608	6.94	C_15_H_14_F_3_N_5_O_2_	354.1168	354.1173	-1.3	337.0907, 319.0801, 307.0801, 281.0645, 97.0396
M335 (BPI-460608)	Hydrolysis	7.73	C_15_H_12_F_3_N_5_O	336.1060	336.1067	-2.1	307.0801, 281.0645, 238.0587, 97.0396, 56.0495
M712	Oxidative defluorination (-F+OH) and glutathione conjugation	8.60	C_28_H_31_F_3_N_8_O_9_S	713.1947	713.1960	-1.8	638.1639, 464.0999, 438.0842, 390.1172, 319.0801, 307.0801, 281.0645
M526	Oxidative defluorination (-F+OH) and cysteine conjugation	8.63	C_21_H_21_F_3_N_6_O_5_S	527.1307	527.1319	-2.3	438.0842, 406.1122, 364.1016, 336.1067, 319.0801, 307.0801, 281.0645, 56.0495
M425	Oxidative defluorination (-F+OH), hydration, and hydrogenation	8.69	C_18_H_18_F_3_N_5_O_4_	426.1381	426.1384	-0.7	319.0801, 307.0801, 281.0645, 238.0587, 146.0812, 56.0495
M528	Cysteine conjugation	8.84	C_21_H_20_F_4_N_6_O_4_S	529.1268	529.1276	-1.5	440.0799, 408.1078, 390.1172, 336.1067, 319.0801, 307.0801, 281.0645, 56.0495
M423 (BPI-460456)	Oxidative defluorination (-F+OH) and hydration	8.96	C_18_H_16_F_3_N_5_O_4_	424.1222	424.1227	-1.3	406.1122, 336.1067, 319.0801, 307.0801, 281.0645, 56.0495
M407-2	Isomer of BPI-460430	8.96	C_18_H_16_F_3_N_5_O_3_	408.1273	408.1278	-1.3	364.1016, 336.1067, 319.0801, 307.0801, 281.0645, 56.0495
M714	Glutathione conjugation	9.04	C_28_H_30_F_4_N_8_O_8_S	715.1911	715.1916	-0.8	640.1601, 440.0799, 408.1078, 390.1172, 336.1067, 319.0801, 281.0645
M453	Dealkylation (-C3H5NO2) and methylation (+CH2) of M526	9.14	C_19_H_18_F_3_N_5_O_3_S	454.1150	454.1155	-1.1	390.1172, 337.0907, 319.0801, 307.0801, 281.0645, 238.0587, 110.0600, 84.0444
M568	Oxidative defluorination (-F+OH), and N-acetylcysteine conjugation	9.18	C_23_H_23_F_3_N_6_O_6_S	569.1420	569.1425	-0.9	438.0842, 406.1122, 390.1172, 336.1067, 319.0801, 307.0801, 281.0645
M552	Defluorination (-F+H) and N-acetylcysteine conjugation	9.53	C_23_H_23_F_3_N_6_O_5_S	553.1467	553.1476	-1.6	422.0893, 390.1172, 319.0801, 307.0801, 281.0645
M407 (BPI-460430)	Defluorination (-F+H) and hydration	9.54	C_18_H_16_F_3_N_5_O_3_	408.1273	408.1278	-1.3	364.1016, 336.1067, 319.0801, 307.0801, 281.0645, 56.0495
M570	N-acetylcysteine conjugation	9.80	C_23_H_22_F_4_N_6_O_5_S	571.1375	571.1382	-1.2	529.1276, 440.0799, 408.1078, 336.1067, 319.0801, 307.0801, 281.0645, 162.0219
M389	Defluorination (-F+H)	10.11	C_18_H_14_F_3_N_5_O_2_	390.1162	390.1173	-2.7	319.0801, 307.0801, 281.0645
M391	Defluorination (-F+H) and hydrogenation	10.17	C_18_H_16_F_3_N_5_O_2_	392.1319	392.1329	-2.6	319.0801, 307.0801, 281.0645, 238.0587

**Note:** ^a^Mass error = (Observed *m/z* – Theoretical *m/z*) / Theoretical *m/z*; 1 ppm = 1 × 10^-6^.

**Table 6:** Relative abundance of MS peak areas of BPI-460372 and its major metabolites after oral administration of BPI-460372 to rats and dogs.

**ID**	**Metabolic Pathway**	**Retention Time**	**Rat Plasma 0-24 h**	**Rat Urine 0-48 h**	**Rat Feces 0-48 h**	**Rat Bile 0-48 h**	**Dog Plasma 0-48 h**
		**(min)**					
BPI-460372	Parent drug	10.65	75.36	0.12	16.95	0.67	30.93
M367	Oxidation (+2O) of BPI-460608	6.53	0.065	2.55	2.77	ND	0.83
M353	Oxidation (+O) and hydrogenation of BPI-460608	6.94	0.16	2.13	2.47	ND	ND
M335 (BPI-460608)	Hydrolysis	7.73	2.48	13.23	4.83	0.070	8.45
M712	Oxidative defluorination (-F+OH) and glutathione conjugation	8.60	ND	ND	ND	0.10	ND
M526	Oxidative defluorination (-F+OH) and cysteine conjugation	8.63	ND	ND	ND	1.52	ND
M425	Oxidative defluorination (-F+OH), hydration, and hydrogenation	8.69	0.14	2.52	2.79	ND	0.98
M528	Cysteine conjugation	8.84	1.53	0.69	1.61	43.88	7.65
M423 (BPI-460456)	Oxidative defluorination (-F+OH) and hydration	8.96	2.71	17.63	9.07	0.58	40.81
M407-2	Isomer of BPI-460430	8.96	ND	0.055	7.88	ND	ND
M714	Glutathione conjugation	9.04	ND	ND	ND	3.27	ND
M453	Dealkylation (-C_3_H_5_NO_2_) and methylation (+CH_2_) of M526	9.14	0.05	17.25	1.57	ND	3.58
M568	Oxidative defluorination (-F+OH), and *N*-acetylcysteine conjugation	9.18	ND	1.80	0.45	3.22	ND
M552	Defluorination (-F+H) and *N*-acetylcysteine conjugation	9.53	0.015	6.73	1.65	0.56	ND
M407 (BPI-460430)	Defluorination (-F+H) and hydration	9.54	16.39	1.02	5.94	0.72	6.23
M570	*N*-acetylcysteine conjugation	9.80	0.12	34.25	3.96	45.40	ND
M389	Defluorination (-F+H)	10.11	0.67	0.050	16.59	0.040	0.15
M391	Defluorination (-F+H) and hydrogenation	10.17	0.35	0.015	21.50	ND	0.36

**Note:** Relative abundance(%)= 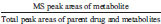 × 100. The relative abundance values shown in the table were the average of male and female samples. ND, not detected by MS.

**Table 7:** The area ratio of BPI-460372 and metabolites in the rat urine after treatment with 20 mg/kg BPI-460372 alone or coadministration with 100 mg/kg AOAA.

**Group**	**BPI-460372**	**M453**	**M528**	**M568**	**M570**	**M335**	**M423**
20 mg/kg BPI-460372	0.89 (54)	0.51 (58)	0.48 (114)	0.16 (71)	0.28 (112)	1.36 (67)	16.6 (71)
20 mg/kg BPI-460372 + 100 mg/kg AOAA	1.48 (69)	0.23 (23)	0.12 (37)	0.06 (20)	0.08 (40)	1.41 (48)	19.0 (57)

**Note:** The data were presented as mean (%CV).

## Data Availability

The data and supportive information are available within the article.

## LIST OF ABBREVIATIONS

AOAA: Aminooxyacetic Acid
AUC: Area Under the Curve
CYP: Cytochrome P450
DDI: Drug-drug Interaction
DMSO: Dimethyl Sulfoxide
E_h_: Hepatic Extraction Ratio
GGT: γ-glutamyl Transpeptidase
GSH: Glutathione
GST: Glutathione-*S*-transferase
HLMs: Human Liver Microsomes
HPLC: High-performance Liquid Chromatography
ICR: Institute of Cancer Research
IS: Internal standard
LC-MS/MS: Liquid Chromatography-tandem Mass Spectrometry
NADPH: Nicotinamide Adenine Dinucleotide Phosphate
NSCLC: Non-small Cell Lung Cancer
PBS: Phosphate Buffer Saline
SD: Sprague-Dawley
TAZ: Transcriptional Coactivator with PDZ-binding Motif
TCA: Tricarboxylic Acid
TCI: Target Covalent Inhibitors
TEAD: Transcriptional Enhanced Associated Domain
TKIs: Tyrosine Kinase Inhibitors
t*_1/2_*: Half-life
YAP: Yes-associated Protein
7-EC: 7-ethoxycoumarin
