# Supplementary material for: In vitro and In Vivo Drug Metabolism Analysis of BPI-460372 - A Covalent TEAD1/3/4 Inhibitor
Source: Curr Drug Metab. 2025 Feb 11;25(10):754–68. doi: 10.2174/0113892002351556250123105344 (PMC12376128; doi:10.2174/0113892002351556250123105344)
Supplement: Supplementary file 1 [file CDM-25-10-754_SD1.pdf]

Supplementary Material

In vitro and *In vivo* Drug Metabolism Analysis of BPI-460372 - A Covalent TEAD1/3/4 Inhibitor

Xiaoyun Liu[1,2], Dafang Zhong[2], Chongzhuang Tang[3], Xiaofeng Xu[1], Hong Lan[1,*] and Xingxing Diao[2,3,*]

[1]*Betta Pharmaceuticals Co., Ltd, Hangzhou, 311100, China;* [2]*Shanghai Institute of Materia Medica, Chinese Academy of Sciences, Shanghai, 201210, China;* [3]*XenoFinder Co., Ltd, Suzhou 215123, China*

1. LC-MS/MS METHODOLOGY

1.1. Metabolic Stability of BPI-460372 in HLMs with or without Specific CYP Inhibitors and Recombinant Human CYP Isoenzyme

BPI-460372 was detected through an XSelect HSS T3 column (2.5 µm, 50 mm × 2.1 mm). The mobile phase contained 0.1% formic acid in water (A) and 0.1% formic acid in acetonitrile (B). The gradient elution program was as follows: 0.00-0.50 min, 30% B; 0.50-1.80 min, 30%-50% B; 1.80-1.85 min, 50%-95% B; 1.85-2.50 min, 95% B; 2.50-2.55 min, 95%-30% B. Then, 30% B from 2.55 to 3.00 min was maintained for equilibration. The flow rate was set to 0.6 mL/min, and the column temperature was kept at 40 °C. The mass spectrometer (MS) was operated in positive ion mode using electrospray ionization (ESI). MS parameters were optimized as follows: 5500 V ion spray voltage, 550 °C probe temperature, 60 ms dwell time, 50 psi ion source gas 1, 50 psi ion source gas 2, and 45 psi curtain gas pressure. Multiple reaction monitoring (MRM) mode was used, in which the ion transitions for BPI-460372 and BPI-460608 (IS) were *m/z* 407.9→318.9 and 336.2→307.2, respectively. The declustering potential (DP) for BPI-460372 and BPI-460608 were 161 V and 96 V, respectively. The collision energies (CE) for BPI-460372 was 33 V and 31 V for BPI-460608.

1.2. Metabolic Stability of BPI-460372BBB in Human, Monkey, Dog, Rat, and Mouse Hepatocytes

BPI-460372 was detected by an ACQUITY UPLC BEH C18 column (1.7 µm, 50 mm × 2.1 mm). The mobile phase contained a mixture of 2 mmol/L ammonium acetate with 0.1% formic acid (A) and 0.1% formic acid in acetonitrile (B). The gradient elution program was as follows: 0.00-0.20 min, 30% B; 0.20-1.60 min, 30%-95% B; 1.60-1.90 min, 95% B; 1.90-2.20 min, 95%-30% B. The flow rate was set to 0.5 mL/min, and the column temperature was kept at 40 °C. The MS was operated in positive ion mode using ESI. MS parameters were optimized as follows: 5500 V ion spray voltage, 550 °C probe temperature, 50 ms dwell time, 60 psi ion source gas 1, 60 psi ion source gas 2, and 40 psi curtain gas pressure. MRM mode was used, in which the ion transitions for BPI-460372 and verapamil (IS) were *m/z* 408.2→319.2 and 455.3→165.2, respectively. The DP for BPI-460372 and verapamil were 170 V and 130 V, respectively. The collision energies (CE) for BPI-460372 was 29 V and 40 V for verapamil.

1.3. The effect of Cysteine *S*-conjugate *β*-lyase inhibitor on the Metabolism of BPI-460372

BPI-460372 and metabolites were separated on a Kinetex C18 column (1.7 µm, 2.1 mm × 100 mm; Phenomenex Corporation, Torrance, CA, USA). 0.1% formic acid in water (A) and 0.1% formic acid in acetonitrile (B) were used as mobile phase. The procedures for gradient elution were as follows: 0.00-1.00 min, 5% B; 1.00-2.00 min, 5%-30% B; 2.00-7.00 min, 30%-80% B; 7.00-7.20 min, 80%-95% B; 7.20-8.00 min, 95%B, and 8.00-8.10 min, 95%-5% B. Then, 5% B from 8.10 to 10.00 min was maintained for equilibration. The flow rate was set at 0.40 mL/min. The samples were maintained at 4°C in an autosampler, and the column temperature was set at 30 °C. The mass spectrometer was operated in positive ion mode using ESI. MS parameters were optimized as follows: ion spray voltage, 5500 V; probe temperature, 500°C; dwell time, 20 ms; ion source gas 1, 55 psi; ion source gas 2, 55 psi; curtain gas, 20 psi; collision gas, 8 psi; entrance potential, 10 V. Verapamil was used as the IS. MRM mode was used, and the ion transitions, DP, CE, and collision cell exit potential (CXP) for these analytes were set as follow.

ID	Q1 Mass (Da)	Q3 Mass (Da)	DP (V)	CE (V)	CXP (V)
BPI-460372	408.1	319.1	126	31	36
M453	454.1	307.1	126	30	20
M528	529.1	440.1	126	20	20
M568	569.1	281.1	126	29	14
M570	571.1	281.1	126	29	14

M335	336.1	307.1	126	31	20
M423	424.1	319.1	126	31	36
verapamil	455.1	165.3	116	39	12

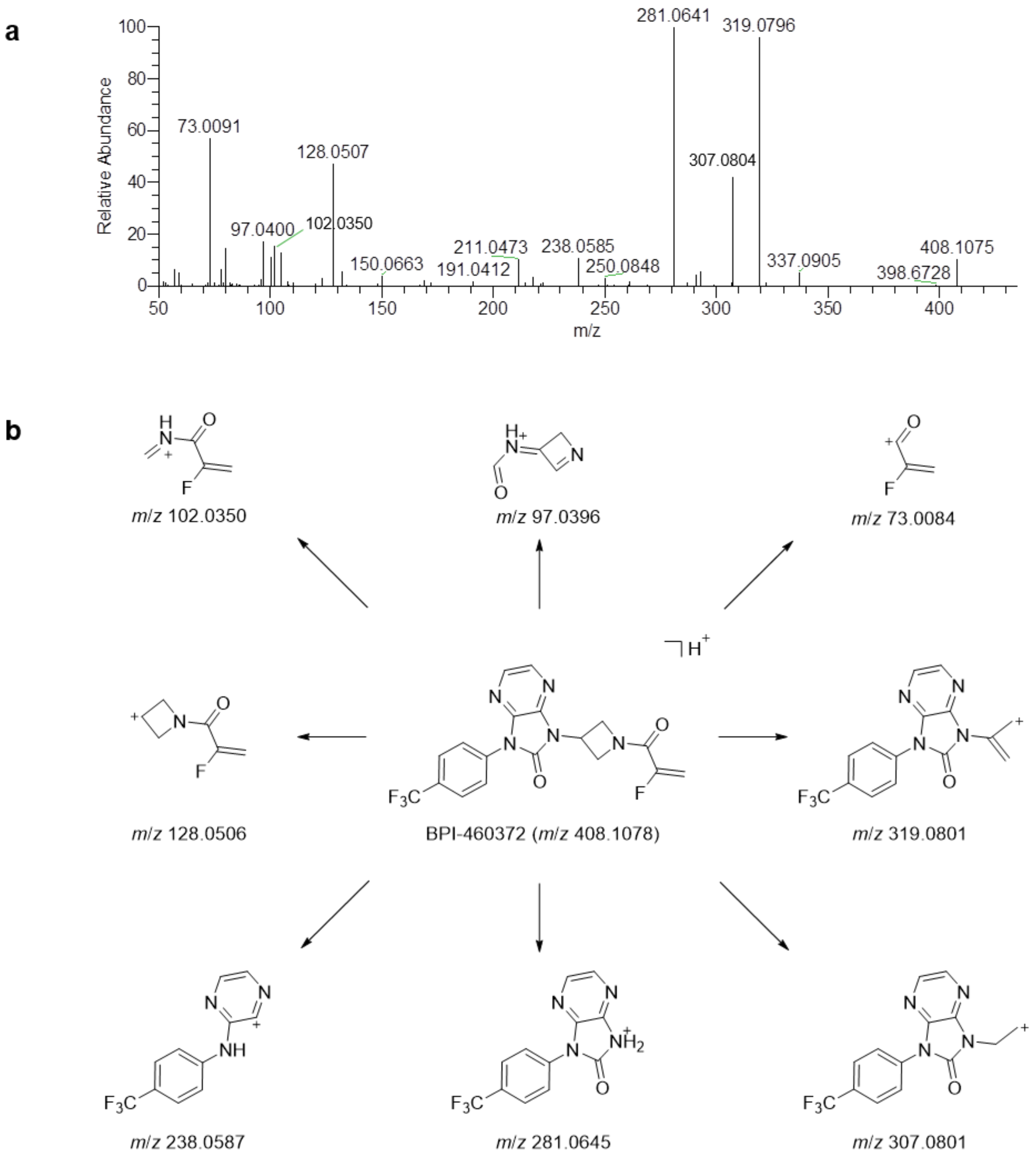

Fig. (S1). The MS/MS product ion spectrum of BPI-460372 (**a**) and the tentative fragmentation patterns (**b**).

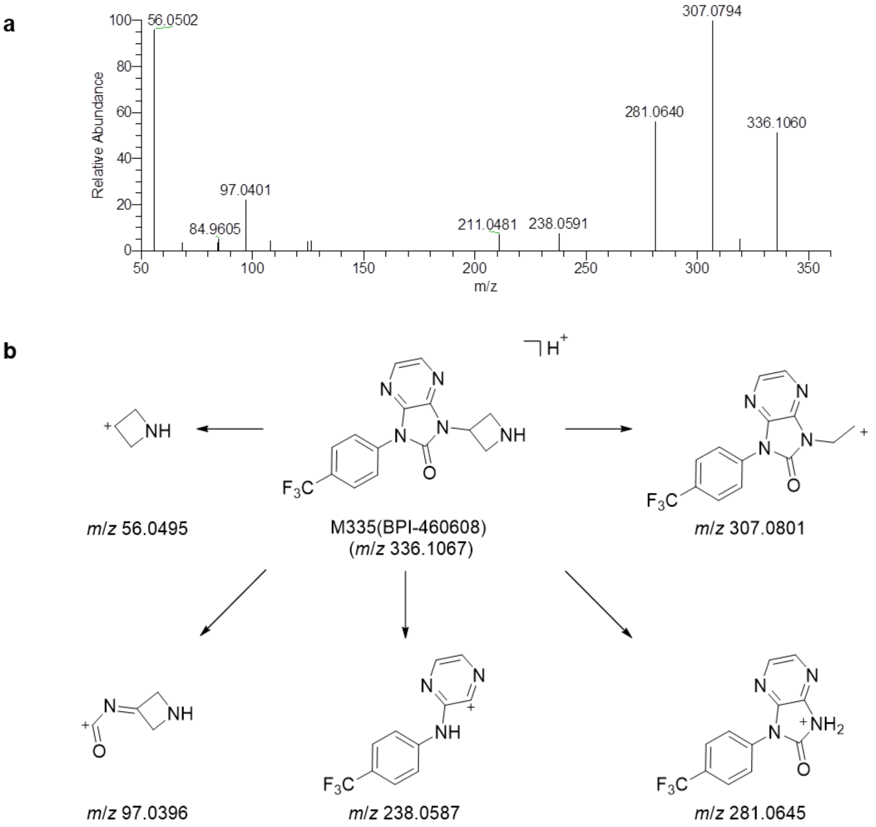

Fig. (S2). The MS/MS product ion spectrum of BPI-460608 (**a**) and the tentative fragmentation patterns (**b**).

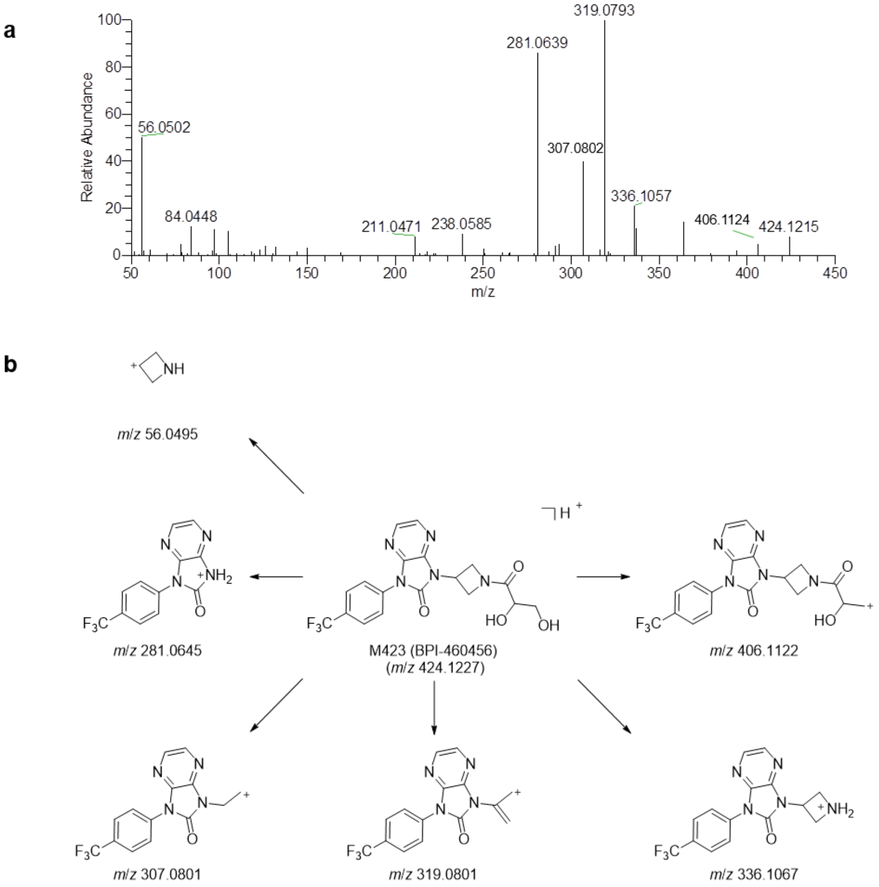

Fig. (S3). The MS/MS product ion spectrum of BPI-460456 (**a**) and the tentative fragmentation patterns (**b**).

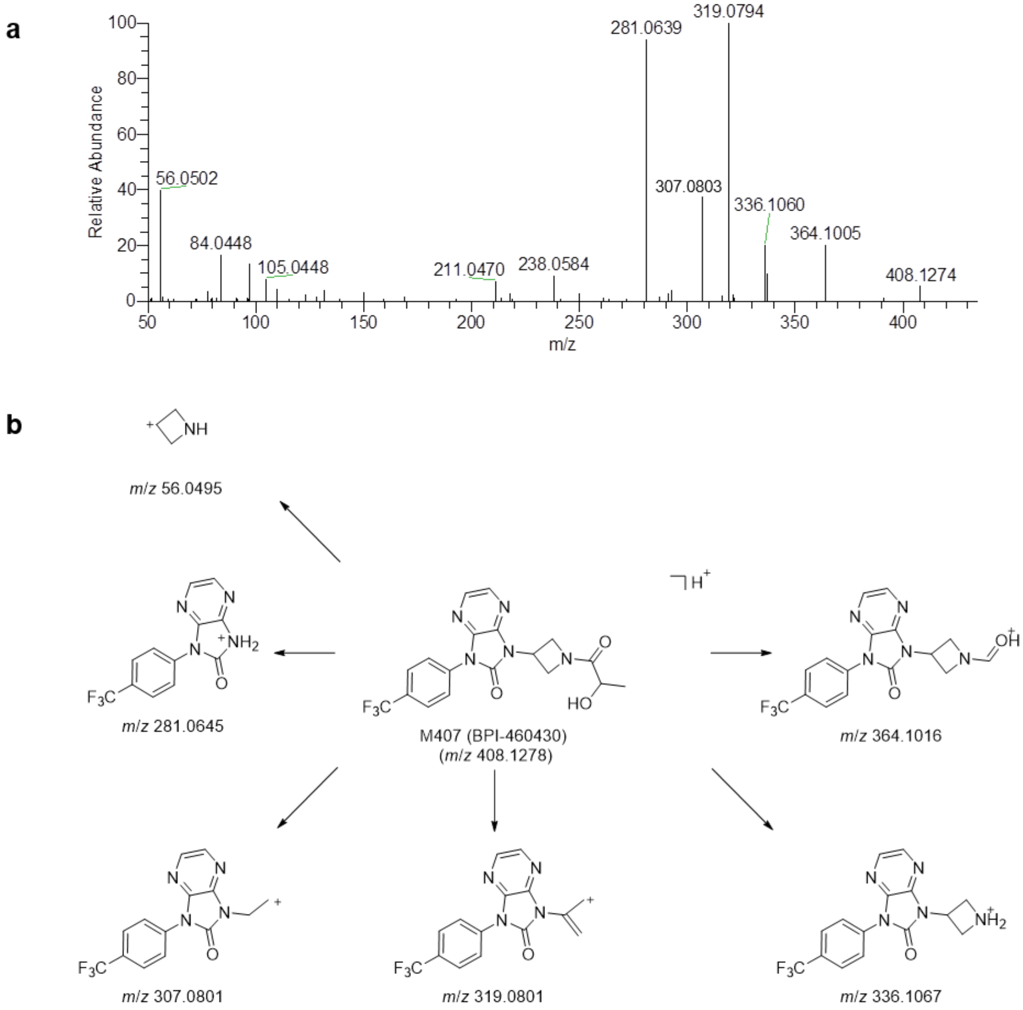

Fig. (S4). The MS/MS product ion spectrum of BPI-460430 (**a**) and the tentative fragmentation patterns (**b**).

Table S1. The inhibition rate of metabolism of positive control substrates in HLM by specific CYP inhibitors and the $CL_{int\ (rCYPj)}$ of positive control substrates through recombinant expressed cytochrome P450 isoforms.

CYP	Positive control substrates	Inhibition rate（%）	$CL_{int\ (rCYPj)}$ ($\mu L*min^{-1}*pmol^{-1}$)
1A2	Phenacetin	79.2	0.151
2B6	Bupropion	75.5	0.0478
2C8	Paclitaxel	78.2	0.457
2C9	Diclofenac	98.2	3.53
2C19	*S*-Mephenytoin	67.4	0.0423
2D6	Dextromethorphan	87.1	NC[a]
3A4	Midazolam	99.7	1.93

Note: [a]Dextromethorphan was completely metabolized in recombinant human CYP2D6 in 5 min, thus K and $CL_{int\ (rCYPj)}$ were not calculated.

Abbreviation: NC, not calculated.

Table S2. Metabolic stability results of testosterone and 7-hydroxycoumarin in human, monkey, dog, rat, and mouse hepatocytes.

Positive control	Species	$t_{1/2}$ (min)	$CL_{int, in virto}$ (µL·min^{-1}·million cells^{-1})	$CL_{int, in vivo}$ (mL·min^{-1}·kg^{-1})	CL_H (mL·min^{-1}·kg^{-1})	E_h
Testosterone	Human	13.3	52.2	188.7	18.7	0.901
	Monkey	11.5	97.1	217.3	36.6	0.832
	Dog	14.1	49.1	337.8	28.3	0.916
	Rat	13.9	121.8	233.5	44.6	0.809
	Mouse	14.9	46.6	554.1	77.4	0.860
7-hydroxycoumarin	Human	20.4	33.9	122.6	17.7	0.856
	Monkey	17.8	39.0	140.6	33.5	0.762
	Dog	29.8	23.3	160.0	25.9	0.838
	Rat	22.5	30.8	144.0	39.9	0.723
	Mouse	49.3	14.1	167.1	58.5	0.650

Table S3. The metabolism of 7-EC in hepatocytes of five species.

Species	7-EC Area Ratio		7-EC-GluA Area Ratio		Proportion of 7-EC remaining in hepato-cytes after 4 h incubation (%)
	T0 h	T4 h	T0 h	T4 h	
Human	3.36E+07	7.50E+06	1.07E+03	3.85E+06	22.32
Monkey	2.65E+07	9.51E+04	6.08E+02	1.55E+07	0.36
Dog	2.18E+07	1.01E+05	0.00E+00	1.27E+07	0.46
Rat	2.64E+07	6.62E+06	1.45E+02	8.08E+05	25.08
Mouse	2.39E+07	1.46E+05	0.00E+00	7.44E+06	0.61

Abbreviation: $T_{0\,h}$, 0 h time point; $T_{4\,h}$, 4 h time point; 7-EC, 7-ethoxycoumarin; 7-EC-GluA, de-ethylation and glucuronidation metabolite of 7-EC.
